# Deep Learning to Improve Breast Cancer Detection on Screening Mammography

**DOI:** 10.1038/s41598-019-48995-4

**Published:** 2019-08-29

**Authors:** Li Shen, Laurie R. Margolies, Joseph H. Rothstein, Eugene Fluder, Russell McBride, Weiva Sieh

**Affiliations:** 1 Icahn School of Medicine at Mount Sinai (ISMMS), Department of Neuroscience, New York, 10029 USA; 2 ISMMS, Department of Diagnostic, Molecular, and Interventional Radiology, New York, 10029 USA; 3 ISMMS, Department of Population Health Science and Policy and Department of Genetics and Genomic Sciences, New York, 10029 USA; 4 ISMMS, Department of Scientific Computing, New York, 10029 USA; 5 ISMMS, Department of Pathology, New York, 10029 USA

**Keywords:** Predictive markers, Computer science, Software, Computational science

## Abstract

The rapid development of deep learning, a family of machine learning techniques, has spurred much interest in its application to medical imaging problems. Here, we develop a deep learning algorithm that can accurately detect breast cancer on screening mammograms using an “end-to-end” training approach that efficiently leverages training datasets with either complete clinical annotation or only the cancer status (label) of the whole image. In this approach, lesion annotations are required only in the initial training stage, and subsequent stages require only image-level labels, eliminating the reliance on rarely available lesion annotations. Our all convolutional network method for classifying screening mammograms attained excellent performance in comparison with previous methods. On an independent test set of digitized film mammograms from the Digital Database for Screening Mammography (CBIS-DDSM), the best single model achieved a per-image AUC of 0.88, and four-model averaging improved the AUC to 0.91 (sensitivity: 86.1%, specificity: 80.1%). On an independent test set of full-field digital mammography (FFDM) images from the INbreast database, the best single model achieved a per-image AUC of 0.95, and four-model averaging improved the AUC to 0.98 (sensitivity: 86.7%, specificity: 96.1%). We also demonstrate that a whole image classifier trained using our end-to-end approach on the CBIS-DDSM digitized film mammograms can be transferred to INbreast FFDM images using only a subset of the INbreast data for fine-tuning and without further reliance on the availability of lesion annotations. These findings show that automatic deep learning methods can be readily trained to attain high accuracy on heterogeneous mammography platforms, and hold tremendous promise for improving clinical tools to reduce false positive and false negative screening mammography results. Code and model available at: https://github.com/lishen/end2end-all-conv.

## Introduction

The rapid advancement of machine learning and especially deep learning continues to fuel the medical imaging community’s interest in applying these techniques to improve the accuracy of cancer screening. Breast cancer is the second leading cause of cancer deaths among U.S. women^1^ and screening mammography has been found to reduce mortality^2^. Despite the benefits, screening mammography is associated with a high risk of false positives as well as false negatives. The average sensitivity of digital screening mammography in the U.S. is 86.9% and the average specificity is 88.9%^3^. To help radiologists improve the predictive accuracy of screening mammography, computer-assisted detection and diagnosis (CAD) software^4^ have been developed and in clinical use since the 1990s. Unfortunately, data suggested that early commercial CAD systems had not led to significant improvement in performance^5–7^ and progress stagnated for more than a decade since they were introduced. With the remarkable success of deep learning in visual object recognition and detection, and many other domains^8^, there is much interest in developing deep learning tools to assist radiologists and improve the accuracy of screening mammography^9–14^. Recent studies^15,16^ have shown that a deep learning based CAD system performed as well as radiologists in standalone mode and improved the radiologists’ performance in support mode.

Detection of subclinical breast cancer on screening mammography is challenging as an image classification task because the tumors themselves occupy only a small portion of the image of the entire breast. For example, a full-field digital mammography (FFDM) image is typically 4000 × 3000 pixels while a potentially cancerous region of interest (ROI) can be as small as 100 × 100 pixels. For this reason, many studies^13,17–21^ have limited their focus to the classification of annotated lesions. Although classifying manually annotated ROIs is an important first step, a fully automated software system must be able to operate on the entire mammogram to provide additional information beyond the known lesions and augment clinical interpretations. If ROI annotations were widely available in mammography databases then established object detection and classification methods such as the region-based convolutional neural network (R-CNN)^22^ and its variants^23–25^ could be readily applied. However, approaches that require ROI annotations^14,26–29^ often cannot be transferred to large mammography databases that lack ROI annotations, which are laborious and costly to assemble. Indeed, few public mammography databases are fully annotated^30^. Other studies^9,10^ have attempted to train neural networks using whole mammograms without relying on any annotations. However, it is hard to know if such networks were able to locate the clinically significant lesions and base predictions on the corresponding portions of the mammograms. It is well known that deep learning requires large training datasets to be most effective. Thus, it is essential to leverage both the few fully annotated datasets, as well as larger datasets labeled with only the cancer status of each image to improve the accuracy of breast cancer classification algorithms.

Pre-training is a promising method to address the problem of training a classifier when the ideal large and complete training datasets are not available. For example, Hinton *et al*.^31^ used layer-wise pre-training to initialize the weight parameters of a deep belief net (DBN) with three hidden layers and then fine-tuned it for classification. They found that pre-training improved the training speed as well as the accuracy of handwritten digit recognition. Another popular training method is to first train a deep learning model on a large database such as the ImageNet^32^ and then fine-tune the model for another task. Although the specific task may not be related to the initial training dataset, the model’s weight parameters are already initialized for recognizing primitive features, such as edges, corners and textures, which can be readily used for a different task. This often saves training time and improves the model’s performance^33^.

In this study, we propose an “end-to-end” approach in which a model to classify local image patches is pre-trained using a fully annotated dataset with ROI information. The patch classifier’s weight parameters are then used to initialize the weight parameters of the whole image classifier, which can be further fine-tuned using datasets without ROI annotations. We used a large public digitized film mammography database with thousands of images to develop the patch and whole image classifiers, and then transferred the whole image classifiers to a smaller public FFDM database with hundreds of images. We evaluated various network designs for constructing the patch and whole image classifiers to attain the best performance. The pipeline required to build a whole image classifier is presented here, as well as the pros and cons of different training strategies.

## Methods

### Converting a classifier from recognizing patches to whole images

**Figure 1:**
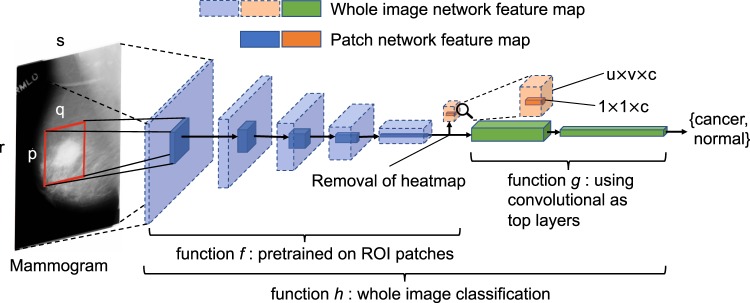
Converting a patch classifier to an end-to-end trainable whole image classifier using an all convolutional design. The function *f* was first trained on patches and then refined on whole images. We evaluated whether removing the heatmap improved information flow from the bottom layers of the patch classifier to the top convolutional layers in the whole image classifier. The magnifying glass shows an enlarged version of the heatmap. This figure is best viewed in color.

To perform classification or segmentation on large complex images, a common strategy involves the use of a classifier in sliding window fashion to recognize local patches on an image to generate a grid of probabilistic outputs. This is followed by another process to summarize the patch classifier’s outputs to give the final classification or segmentation result. Such methods have been used to detect metastatic breast cancer using whole slide images of sentinel lymph node biopsies^34^ and to segment neuronal membranes in microscopic images^35^. However, this strategy requires two steps that each needs to be optimized separately. Here, we propose a method to combine the two steps into a single step for training on the whole images (Fig. 1). Assume we have an input patch $X\in {{\rm{IR}}}^{{\rm{p}}\times {\rm{q}}}$ and a patch classifier which is a function *f* so that $f(X)\in {{\rm{IR}}}^{{\rm{c}}}$, where the function’s output satisfies *f*(*X*)_*i*_ ∈ [0, 1] and ${{\rm{\Sigma }}}_{i=1}^{c}f{(X)}_{i}=1$ and *c* is the number of classes of the patches. Here, *c* = 5 and the classes are: benign calcification, malignant calcification, benign mass, malignant mass and background for each patch from a mammogram. Assume the input patch is extracted from an image $M\in {{\rm{IR}}}^{{\rm{r}}\times {\rm{s}}}$ where *p* ≪ *r* and *q* ≪ *s*. If the function *f* represents a convolutional neural network (CNN), then *f* can be applied to *M* without changing the network parameters so that $f(M)\in {{\rm{IR}}}^{{\rm{u}}\times {\rm{v}}\times {\rm{c}}}$, where *u* > 1 and *v* > 1 depend on the image size and the stride of the patch classifier. This is possible because of the weight sharing and locality properties of a CNN^36^. If the function *f* represents a different class of neural networks, such as the multilayer perceptron (MLP), then this becomes infeasible because a MLP requires the input to be fixed. Therefore, after changing the input from *X* to *M*, we have a *u* × *v* grid of probabilistic outputs of *c* classes (referred to as “heatmap”) instead of a single output of *c* classes. Hence the heatmap has a size of *u* × *v* × *c*. More layers can then be added on top of the heatmap to transform the outputs and connect with the final classification output of the image. Adding a convolutional layer on top of the patch classifier’s outputs turns the entire patch classifier into a filter and enlarges its receptive field. For example, if the patch classifier has a receptive field of 224 × 224 with a stride = 32, adding a 3 × 3 convolutional layer on top of it increases each side of the receptive field to 224 + (3 − 1) × 32 = 228. Thus, the top layers effectively use the patch classifier to “scan” the whole image, looking for cues of cancerous lesions and extracting higher level features that can finally be used for whole image classification. Using function *g* to represent the top layers, the whole image classification function can be written as $h(M)=g(f(M))\in {{\rm{IR}}}^{{\rm{d}}}$, where *d* is the number of classes of the whole image. Typically, *d* = 2 represents the two classes we want to predict: malignant and nonmalignant (benign or normal).

The function *h* accepts whole images as input and produces labels at the whole image level. Therefore, it is end-to-end trainable, providing two advantages over the two-step approach. First, the entire network can be jointly trained, avoiding sub-optimal solutions from each step; Second, the trained network can be transferred to another dataset without explicit reliance on ROI annotations. Large mammography databases with ROI annotations are rare and expensive to obtain. The largest public database with ROI annotations for digitized film mammograms – DDSM^37^ – contains several thousand images with pixel-level annotations, which can be exploited to train a patch classifier *f*. Once the patch classifier is converted into a whole image classifier *h*, it can be fine-tuned on other databases using only image-level labels. This approach allows us to significantly reduce the requirement for ROI annotations, and has many applications in medical imaging in addition to breast cancer detection on screening mammograms.

### Network design

A modern CNN is typically constructed by stacking convolutional layers on top of the input, followed by one or more fully connected (FC) layers to join with the classification output. Max pooling layers are often used amid convolutional layers to improve translational invariance and to reduce feature map size. In this study, two popular CNN structures are compared: the VGG network^38^ and the residual network (Resnet)^39^. Consecutive network layers can be naturally grouped into “blocks” so that the feature map size is reduced (typically by a factor of 2) either at the beginning or at the end of a block but stays the same elsewhere in the block. For example, a “VGG block” is a stack of several 3 × 3 convolutional layers with the same depth followed by a 2 × 2 max pooling layer that reduces the feature map size by a factor of 2. Although other filter sizes can be used, 3 × 3 convolution and 2 × 2 max pooling are widely used, and employed throughout this study unless otherwise stated. Therefore, a VGG block can be represented by the pattern of *N* × *K*, where *N* represents the depth of each convolutional layer and *K* represents the number of convolutional layers. A “Resnet block” uses stride = 2 in the first convolutional layer instead of 2 × 2 max pooling to reduce feature map size at the beginning of the block, followed by the stacking of several convolutional layers. We use the “bottleneck design^39^” which consists of repeated units of three convolutional layers that have filter sizes of 1 × 1, 3 × 3 and 1 × 1, respectively. A key feature of the Resnet block is that a shortcut is made between the two ends of each unit so that the features are directly carried over and therefore each unit can focus on learning the “residual” information^39^. Batch normalization (BN) is used in every convolutional layer in the Resnet, which is known to speedup convergence and also has a regularization effect^40^. A Resnet block can be represented by the pattern of [*L* − *M* − *N*] × *K*, where *L*, *M* and *N* represent the depths of the three convolutional layers in a unit and *K* represents the number of units. Here, the 16-layer VGG network (VGG16) and the 50-layer Resnet (Resnet50) are used as patch classifiers. The original design of the VGG16^38^ consisted of five VGG blocks followed by two FC layers. To be consistent with the Resnet50, we replaced the two FC layers with a global average pooling layer which calculates the average activation of each feature map for the output of the last VGG block. For example, if the output of the last VGG block has a size of 7 × 7 × 512 (height × width × channel), after the global average pooling layer the output becomes 512. This output is then connected to the classification output with a FC layer.

A straightforward approach to construct a whole image classifier from a patch classifier involves flattening the heatmap and connecting it to the image’s classification output using FC layers. To increase the model’s translational invariance to the patch classifier’s output, a max pooling layer can be used after the heatmap. Further, a shortcut can be made between the heatmap and the output to make the training easier. The heatmap results directly from the patch classifier’s output which uses the softmax activation:

1 $$f{({\bf{z}})}_{j}=\frac{{e}^{{z}_{j}}}{{{\rm{\Sigma }}}_{i=1}^{c}{e}^{{z}_{i}}}\,{\rm{for}}\,j=1,\ldots ,c$$

However, the softmax activation diminishes gradients for large inputs, which may impede gradient flow when it is used in an intermediate layer. Therefore, the rectified linear units (ReLU) can be used instead:

2 $$f{({\bf{z}})}_{j}=max(0,{z}_{j})\,{\rm{for}}\,j=1,\ldots ,c$$

In the following, when we refer to the heatmap in a whole image classifier, the activation is always assumed to be ReLU unless otherwise stated.

We further propose to use convolutional layers as top layers, which preserve spatial information. Two blocks of convolutional layers (VGG or Resnet) can be added on top of the patch classifier layers, followed by a global average pooling layer and then the image’s classification output (Fig. 1). Therefore, this design creates an “all convolutional” network for whole image classification. As Fig. 1 shows, the heatmap abruptly reduces the depth of the feature map between the patch classifier layers and the top layers, which may cause information loss in the whole image classification. Therefore, we also evaluated the results when the heatmap is removed entirely from the whole image classifier to allow the top layers to fully utilize the features extracted from the patch classifier.

### Computational environment

All experiments in this study were carried out on a Linux workstation equipped with an NVIDIA 8 GB Quadro M4000 GPU card.

## Results

### Developing patch and whole image classifiers on CBIS-DDSM

#### Setup and processing of the dataset

The DDSM^37^ contains digitized film mammograms in a lossless-JPEG format that is now obsolete. We used a later version of the database called CBIS-DDSM^41^ which contains images that are converted into the standard DICOM format. The dataset which consisted of 2478 mammography images from 1249 women was downloaded from the CBIS-DDSM website, and included both craniocaudal (CC) and mediolateral oblique (MLO) views for most of the exams. Each view was treated as a separate image in this study. We randomly split the CBIS-DDSM dataset 85:15 at the patient level to create independent training and test sets. The training data was further split 90:10 to create an independent validation set. The splits were done in a stratified fashion to maintain the same proportion of cancer cases in the training, validation and test sets. The total numbers of images in the training, validation and testing sets were: 1903, 199 and 376, respectively.

The CBIS-DDSM database contains the pixel-level annotations for the ROIs and their pathologically confirmed labels: benign or malignant. It further labels each ROI as a calcification or mass. Most mammograms contained only one ROI. All mammograms were converted into PNG format and downsized to 1152 × 896 using interpolation; no image cropping was performed. The downsizing was motivated by the limitation of GPU memory size. Two patch datasets were created by sampling image patches from ROIs and background regions. All patches had the same size of 224 × 224, which were large enough to cover most of the ROIs annotated. The first dataset (S1) consisted of sets of patches in which one was centered on the ROI and one is a random background patch from the same image. The second dataset (S10) consisted of 10 patches randomly sampled from around each ROI, with a minimum overlapping ratio of 0.9 with the ROI and inclusion of some background, to more completely capture the potentially informative region; and an equal number of background patches from the same image. All patches were classified into one of the five categories: background, malignant mass, benign mass, malignant calcification and benign calcification.

#### Network training

Training a whole image classifier was achieved in two steps. The first step was to train a patch classifier. We compared the networks with pre-trained weights using the ImageNet^32^ database to those with randomly initialized weights. In a pre-trained network, the bottom layers represent primitive features that tend to be preserved across different tasks, whereas the top layers represent higher-order features that are more related to specific tasks and require further training. Using the same learning rate for all layers may destroy the features that were learned in the bottom layers. To prevent this, a 3-stage training strategy was employed in which the parameter learning is frozen for all but the final layer and progressively unfrozen from the top to the bottom layers, while simultaneously decreasing the learning rate. The 3-stage training strategy on the S10 patch set was as follows:

1. Set learning rate to 10^−3^ and train the last layer for 3 epochs.
2. Set learning rate to 10^−4^, unfreeze the top layers and train for 10 epochs, where the top layer number is set to 46 for Resnet50 and 11 for VGG16.
3. Set learning rate to 10^−5^, unfreeze all layers and train for 37 epochs for a total of 50 epochs.

In the above, an epoch was defined as a sweep through the training set. For the S1 patch dataset, the total number of epochs was increased to 200 because it was much smaller and less redundant than the S10 patch dataset. For randomly initialized networks a constant learning rate of 10^−3^ was used. Adam^42^ was used as the optimizer and the batch size was set to be 32. The sample weights were adjusted within each batch to balance the five classes.

The second step was to train a whole image classifier converted from the patch classifier (Fig. 1). A 2-stage training strategy was employed to first train the newly added top layers (i.e. function *g*) and then train all layers (i.e. function *h*) with a reduced learning rate, which was as follows:

1. Set learning rate to 10^−4^, weight decay to 0.001 and train the newly added top layers for 30 epochs.
2. Set learning rate to 10^−5^, weight decay to 0.01 and train all layers for 20 epochs for a total of 50 epochs.

We found that the VGG-based image classifiers showed sign of continuing improvement towards the end of the 50 epochs, while the Resnet-based image classifiers had already converged. To be fair for the VGG-based image classifiers, we continued to train them with 200 additional epochs. Due to GPU memory limits, a batch size of 2 was used.

The average gray scale value of the whole image training set was subtracted from both patch and whole image datasets in training. No other preprocessing was applied. To improve the generalization of final models, data augmentation was performed using the following random transformations: horizontal and vertical flips, rotation in [−25, 25] degrees, zoom in [0.8, 1.2] ratio and intensity shift in [−20, 20] pixel values.

#### Development of patch classifiers

Table 1 shows the accuracy of the classification of image patches into 5 classes using Resnet50 and VGG16 in the CBIS-DDSM test set. A bootstrapping method with 3000 runs was used to derive the 95% confidence intervals for patch classification accuracy. The S10 set was more difficult to classify than the S1 set because it contained patches sampled from around ROIs, rather than centered on the ROI, that were more difficult to distinguish from background regions. On the S1 set, both randomly initialized and pre-trained Resnet50 classifiers achieved similar accuracy but the pre-trained network converged after half as many epochs as the randomly initialized one. On the S10 set, the pre-trained Resnet50 outperformed the randomly initialized one by a large margin, achieving an accuracy [95% confidence interval (CI)] of 0.89 [0.88, 0.90]. These results showed that pre-training can greatly help network convergence and performance. Therefore, pre-trained networks were used for the rest of the study. The accuracy of the pre-trained VGG16 (0.84 [0.83, 0.85]) on the S10 set was lower than that of the pre-trained Resnet50.

**Table 1:** Accuracy of the patch classifiers using the Resnet50 and VGG16 in the independent test set.

Model	Pretrained	Patch set	Accuracy	#Epochs
Resnet50	N	S1	0.97 [0.96, 0.98]	198
Resnet50	Y	S1	0.99 [0.98, 1.00]	99
Resnet50	N	S10	0.63 [0.62, 0.64]	24
Resnet50	Y	S10	0.89 [0.88, 0.90]	39
Resnet50	Y	S1g	0.76 [0.74, 0.79]	84
VGG16	Y	S10	0.84 [0.83, 0.85]	25

#Epochs indicates the epoch when the highest accuracy was reached in the validation set.

**Figure 2:**
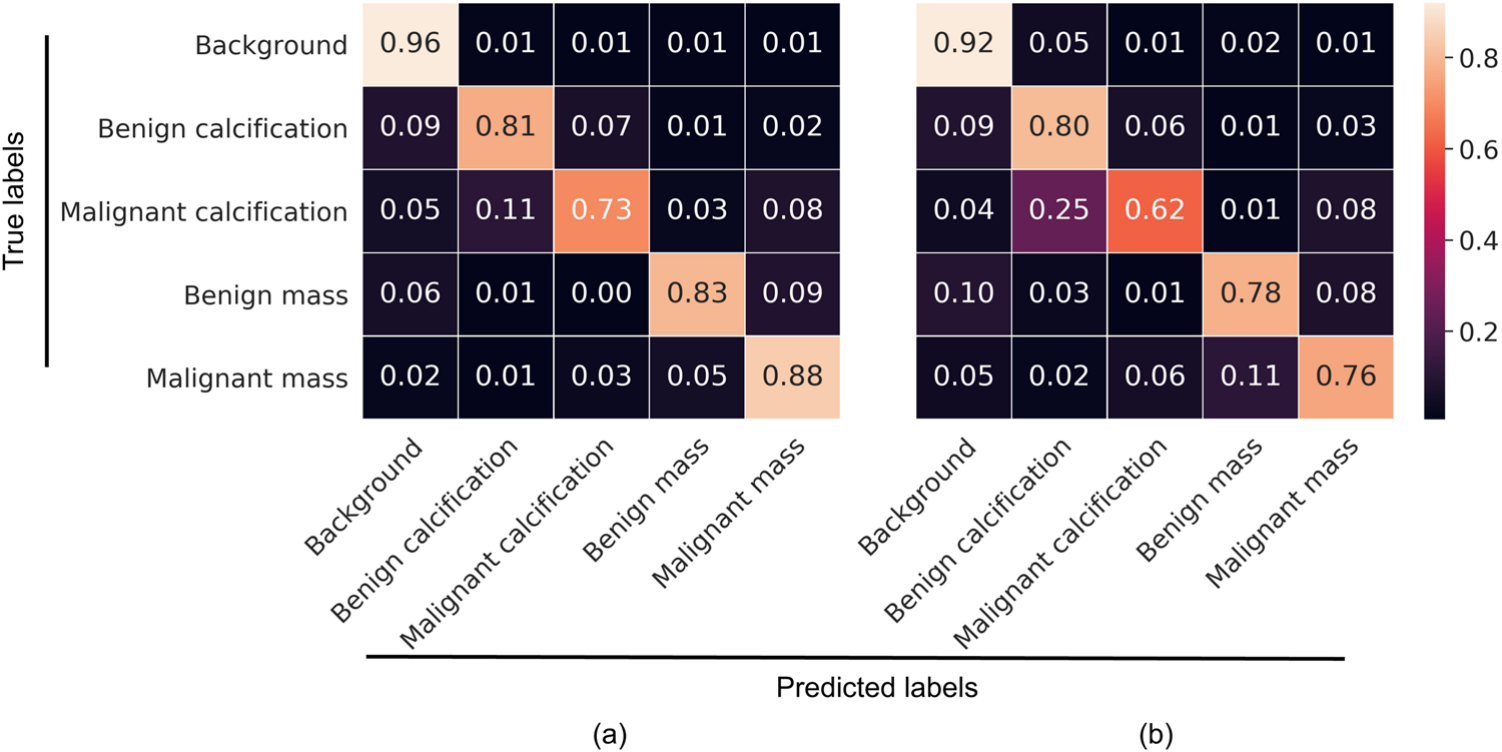
Confusion matrix analysis of 5-class patch classification for Resnet50 (**a**) and VGG16 (**b**) in the S10 test set. The matrices are normalized so that each row sums to one. This figure is best viewed in color.

To further characterize performance, confusion matrix analyses were conducted on the Resnet50 and VGG16 patch classifiers in the S10 test set (Fig. 2). For both patch classifiers, all five classes were predicted into the correct categories with the highest probability. The background class was easiest, and malignant calcifications hardest to classify. Malignant calcifications were most likely to be misclassified as benign calcification, followed by malignant mass. Benign calcifications were most likely to be misclassified as background, followed by malignant calcification. Malignant masses were most likely to be misclassified as benign masses, while benign masses were most likely to be misclassified as malignant masses or background, depending on the patch classifier.

#### Converting patch to whole image classifiers

Using pre-trained Resnet50 and VGG16 patch classifiers, we tested several different configurations for the top layers of the whole image classifiers. We also evaluated removal of the heatmap and adding two Resnet or VGG blocks on top of the patch classifier layers, followed by a global average pooling layer and the classification output. Model performance was assessed by computing the per-image AUCs on the independent test set.

Resnet-based networks: To evaluate whether the patch classifiers trained on the S1 and S10 datasets are equally useful for whole image classification, the Resnet50 patch classifiers were used. In the original design of the Resnet50^39^, *L* ≡ *M*, *N* is four times *L* and *K* is 3 or more; the *L* of the current block is also double of the *L* of the previous block. However, we found this design to exceed our GPU memory limit when it is used for the top layers of the whole image classifier. In the initial experiments, we used instead the same configuration of [512 − 512 − 2048] × 1 for two Resnet blocks on top of the patch classifier. A bootstrapping method with 3000 runs was used to derive 95% confidence intervals for AUCs and AUC differences. The whole image classifier trained using the S10 set (mean AUC = 0.85) performed much better than that trained using the S1 set (mean AUC = 0.63) (Table 2), despite its poorer patch classification accuracy (Table 1). The S10 dataset contains more information about the ROIs as well as their adjacent regions and other background regions on the image than the S1 dataset, which allows a patch classifier to extract more features that can be important for whole image classification. To test this hypothesis, we created another patch set (referred to as S1g) with one patch each from the ROI and background but a large patch size of 448 × 448 to include the surrounding area. The patch classification accuracy in S1g was much lower than that in S10 (Table 1). However, the image classification accuracy was similar for models trained on S1g and S10 (Table 2) with an estimated AUC difference [95% confidence interval] of −0.023 [−0.061, 0.016] supporting the hypothesis that the background regions contain useful information. For the rest of the study, only patch classifiers trained on the S10 dataset were used. Varying the configuration by using two Resnet blocks of [512 − 512 − 1024] × 2 yielded a mean AUC of 0.86, while reducing the depths and *K* of the two Resnet blocks to: [256 − 256 − 256] × 1 and [128 − 128 − 128] × 1 did not significantly decrease the AUC (Table 2). This result showed that the depths of the Resnet blocks were relatively uncorrelated with the performance of the whole image classifiers.

**Table 2:** Per-image AUCs of whole image classifiers using the Resnet50 as patch classifiers in the independent test set.

Patch set	Block 1	Block 2	AUC [95% CI]	A-AUC [95% CI]	#Epochs
S1	[512-512-2048] × 1	[512-512-2048] × 1	0.63 [0.58, 0.67]	NA	35
S1g	[512-512-2048] × 1	[512-512-2048] × 1	0.83 [0.79, 0.86]	NA	38
S10	[512-512-2048] × 1	[512-512-2048] × 1	0.85 [0.82, 0.88]	0.86 [0.83, 0.89]	20
**S10**	**[512**-**512**-**1024]** × **2**	**[512**-**512**-**1024]** × **2**	**0**.**86 [0**.**83**, **0**.**89]**	**0**.**87 [0**.**83**, **0**.**90]**	**34**
S10	[256-256-256] × 1	[128-128-128] × 1	0.84 [0.81, 0.87]	0.86 [0.82, 0.89]	25
**S10**	**256** × **1**	**128** × **1**	**0**.**87 [0**.**84**, **0**.**90]**	**0**.**88 [0**.**84**, **0**.**90]**	**36**
**Insert heatmap between patch classifier and top layers**					
S10	[512-512-1024] × 2	[512-512-1024] × 2	0.80 [0.76, 0.84]	NA	47
S10	[64-64-256] × 2	[128-128-512] × 2	0.81 [0.77, 0.85]	NA	41
**Add heatmap and fully connected** (**FC**) **layers on top** (**S10 patch set**)					
**Pool size**	**FC1**	**FC2**			
5 × 5	64	32	0.74 [0.69, 0.78]	NA	28
2 × 2	512	256	0.72 [0.67, 0.76]	NA	47
1 × 1	2048	1024	0.65 [0.60, 0.69]	NA	43

#Epochs indicates the epoch when the highest AUC was reached in the validation set. The best performing models are shown in boldface.

VGG-based networks: We tested whole image classifiers using VGG16 as the patch classifier and VGG blocks as the top layers. BN was used for the VGG blocks on the top except for the VGG16 patch classifier because it is a pre-trained network which cannot be modified. The VGG-based whole image classifiers performed similarly to the Resnet-based ones but took longer to achieve the same performance level (Table 3). In contrast to the Resnet, using more convolutional layers and higher depths in VGG blocks led to poorer performance: using two VGG blocks of 256 × 1 and 128 × 1 (mean AUC = 0.85) performed better than two VGG blocks of 512 × 3 (mean AUC = 0.81) with an AUC difference of 0.041 [0.011, 0.071]. Reducing the depths further to 128 and 64 did not further improve the AUC. This result illustrates that controlling model complexity (i.e., #layers and depths) is important for achieving good performance with the VGG-based networks, which are more likely to overfit.

**Table 3:** Per-image AUCs of whole image classifiers using the VGG16 as patch classifiers in the independent test set.

Patch set	Block 1	Block 2	AUC [95% CI]	A-AUC [95% CI]	#Epochs
S10	512 × 3	512 × 3	0.81 [0.77, 0.84]	0.82 [0.78, 0.85]	91
**S10**	**256** × **1**	**128** × **1**	**0**.**85 [0**.**81**, **0**.**88]**	**0**.**86 [0**.**83**, **0**.**89]**	**61**
S10	128 × 1	64 × 1	0.84 [0.80, 0.87]	0.86 [0.82, 0.89]	142
**S10**	**[512**-**512**-**1024]** × **2**	**[512**-**512**-**1024]** × **2**	**0**.**85 [0**.**82**, **0**.**88]**	**0**.**88**, **[0**.**85**, **0**.**91]**	**165**
**Add heatmap and fully connected** (**FC**) **layers on top** (**S10 patch set**)					
**Pool size**	**FC1**	**FC2**			
5 × 5	64	32	0.71 [0.66, 0.75]	NA	26
2 × 2	512	256	0.68 [0.63, 0.73]	NA	27
1 × 1	2048	1024	0.70 [0.65, 0.74]	NA	50

#Epochs indicates the epoch when the highest AUC was reached in the validation set. The best performing models are shown in boldface.

Hybrid networks: We also created two “hybrid” networks by adding the VGG top layers that performed the best (two VGG blocks of 256 × 1 and 128 × 1) on top of the Resnet50 patch classifier; and the Resnet top layers that performed the best (two Resnet blocks of the same configuration of [512 − 512 − 1024] × 2) on top of the VGG16 patch classifier. The two hybrid networks achieved mean AUCs of 0.87 and 0.85, respectively, and were among the best performing models (Tables 2 and 3).

**Figure 3:**
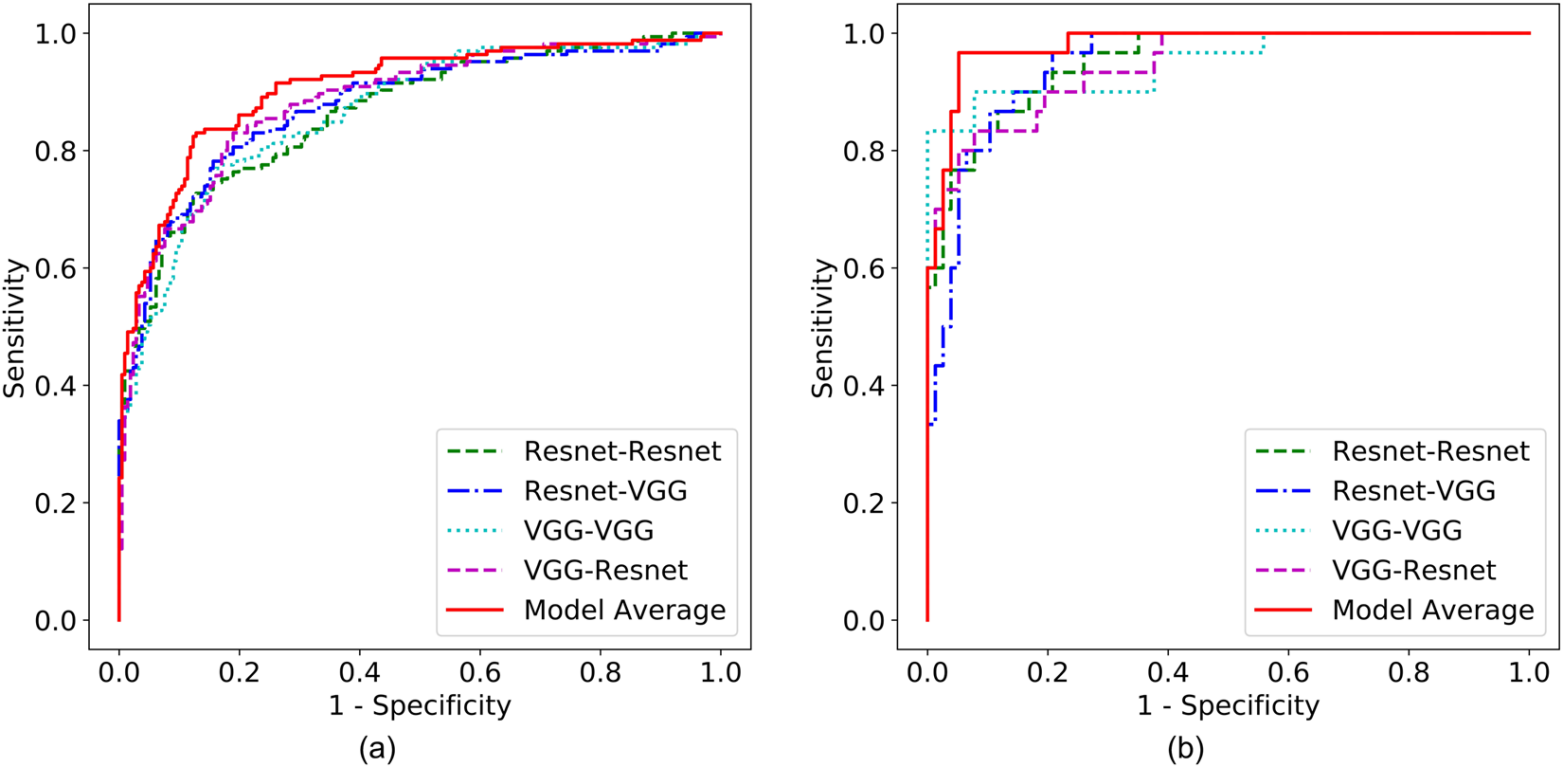
ROC curves for the four best individual models and ensemble model on the CBIS-DDSM (**a**) and INbreast (**b**) test sets. This figure is best viewed in color.

Augmented prediction and model averaging: Augmented prediction was implemented by horizontally and vertically flipping an image to obtain four images and taking an average of the four images’ scores. This technique increased the AUC (referred to as A-AUC) for each model by 0.01–0.03 (Tables 2 and 3), although only some of the models showed significant increase based on the 95% confidence intervals of AUC differences (Table S1). The four best performing models were combined into an ensemble model by taking the average of their augmented prediction scores. Two of the four best models used Resnet50 and VGG16 as patch classifiers and Resnet and VGG blocks as top layers, respectively (referred to as Resnet-Resnet and VGG-VGG); and the remaining two were hybrid models (referred to as Resnet-VGG and VGG-Resnet). Figure 3a shows the Receiver Operating Characteristic (ROC) curves of the four best models and the ensemble model, which yielded an AUC of 0.91. Because the clinical significance of a false negative (FN) is higher than that of a false positive (FP), a clinically useful system should not have significantly lower sensitivity than the current standard of care. Therefore, we evaluated model performance using a sensitivity of 86% as a benchmark based upon the estimated average for U.S. radiologists^3^, and determined the model specificity to be 80.1% at a similar sensitivity of 86.1%.

**Figure 4:**
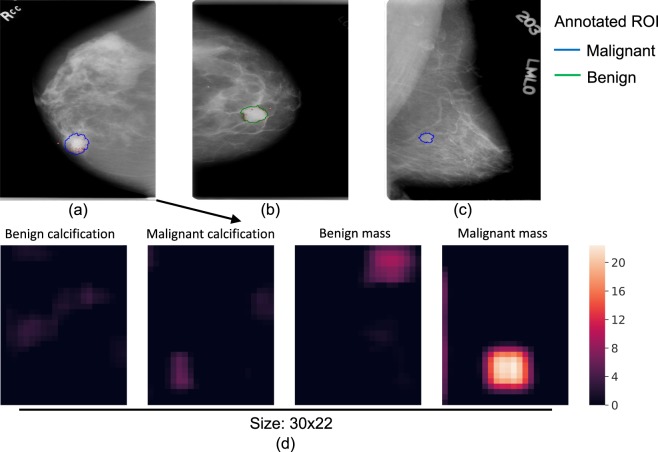
Saliency maps of TP (**a**), FP (**b**) and FN (**c**) image classifications. The outlines represent the regions of interest annotated by the radiologist, and biopsy-confirmed to contain either malignant (blue) or benign (green) tissue. The red dots represent the gradients of the input image with respect to the cancer class output. The gradients were rescaled to be within [0, 1] and a low cutoff of 0.06 was used to remove background noise. Heatmaps (**d**) of the four non-background classes for input image (**a**). The colors of the heatmaps represent the activation values after ReLU. This figure is best viewed in color.

Saliency map and error analysis: Saliency maps were created using the Resnet-VGG model (Fig. 4a–c), which showed the gradients of the input image with respect to the cancer class output. We used the guided back-propagation approach^43^ that calculates only positive gradients for positive activations. A saliency map illustrates which area of the input image is considered to be responsible for the cancer prediction by a whole image classifier. Figure 4a shows the saliency map of a true positive (TP) image where the identified area is in or close to the malignant ROI. This shows that the image classifier was able to correctly locate the cancerous region on which its decision was based. Figure 4b shows a typical FP image where the identified region is located in a benign ROI that resembles a malignant ROI. Figure 4c shows a typical FN image where the malignant ROI is difficult to discern and no response passes the low cutoff.

Combining the CC and MLO views for prediction: Combining the CC and MLO views may increase performance because each view can contain unique information. After removing the samples where only a single view was available, 90% of the test set remained for analysis of both views from each of 169 breasts. We used a simple approach of taking the average score of the two views. A breast-level bootstrapping method was used (3000 runs) to compare two-view vs. single-view AUCs for the four best models above: Resnet-Resnet, Resnet-VGG, VGG-VGG and VGG-Resnet. The mean AUC differences were 0.030 [0.018, 0.042], 0.027 [0.016, 0.037], 0.040 [0.028, 0.051] and 0.048 [0.032, 0.064], respectively. Thus, using two views when available significantly increased the AUCs in comparison to single views for all the models tested here.

Max-pooling, shortcut and FC layers: We tested an alternative design by using the heatmap followed by a max-pooling and two FC layers, including a shortcut between the heatmap and the classification output. The Resnet50 and VGG16 patch classifiers were used. The FC layer sizes were chosen to gradually reduce the layer outputs. When the pooling size increased from 1 × 1 (i.e. no pooling) to 5 × 5, the AUCs did not show significant changes with the exception of pooling size 1 × 1 for the Resnet50 patch classifier, in which case the AUC was significantly lower than the others (Tables 2 and 3). The best mean AUC for these models was 0.74, falling short of the performance of the all convolutional models.

Evaluation of the heatmap in all convolutional networks: To test our hypothesis that the heatmap can cause information loss in the whole image classification network, we inserted a heatmap in the Resnet-based whole image classifier with two [512 − 512 − 1024] × 2 blocks as top layers. The heatmap inserted was a 1 × 1 convolutional layer that reduces the number of filters from the previous convolutional layer (2048) to 5, which corresponds to the 5 classes of the patch classifier. To facilitate the back-propagation of gradients, ReLU was used to replace the softmax activation in the heatmap. Figure 4d shows an example heatmap that provides a rough segmentation of the input image; the top layers then use the segmentation to classify the whole image. This model achieved a mean AUC of 0.80 (Table 2), which was significantly lower than that of the same classifier without the heatmap with an AUC difference of −0.050 [−0.088, −0.012]. To exclude the possibility that the top layers were overfit due to the shallow depth of the heatmap, another model with reduced complexity using two Resnet blocks of [64 − 64 − 256] × 2 and [128 − 128 − 512] × 2 was tested, which achieved a similar mean AUC of 0.81 (AUC difference of −0.044 [−0.075, −0.012]). These results indicated that removing the heatmap was beneficial to the whole image classification networks.

Comparison to prior two-step approach: Finally, for comparison we tested a previously reported approach^34^ that used a probability cutoff to binarize the heatmap into a binary image that represents each pixel as background (0) or ROI (1). This was repeated for each of the 4 foreground classes. We then extracted regional features (such as area, major axis length and mean intensity) from the ROIs of the binary images and trained a random forest classifier (#trees = 500, max depth = 9, min samples split = 300) on the regional features. The Resnet50 patch classifier was used and the softmax activation was used in the heatmap to obtain the probabilities for the 5 classes. Four cutoffs—0.3, 0.5, 0.7 and 0.9 were used to binarize the heatmaps and the regional features were combined. This approach achieved an AUC of 0.73, and was inferior to the all convolutional models.

### Transfer learning for whole image classification on INbreast

#### Setup and processing of the dataset

**Figure 5:**
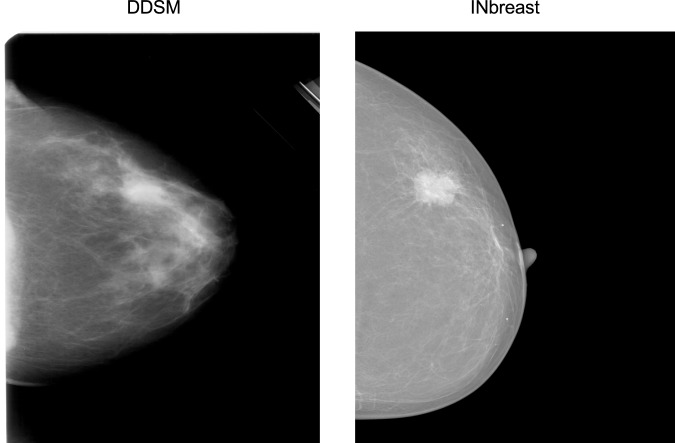
Representative examples of a digitized film mammogram from CBIS-DDSM and a digital mammogram from INbreast.

The INbreast^30^ dataset is a public database containing more recently acquired FFDM images. These images have different intensity profiles compared with digitized film mammograms from the CBIS-DDSM, as illustrated by example images from the two databases (Fig. 5). Therefore, INbreast provides an excellent opportunity to test the transferability of a whole image classifier across mammography platforms. The INbreast database contains 115 patients and 410 mammograms including both CC and MLO views. We analyzed each view separately like above. The INbreast database includes radiologists’ BI-RADS^44^ assessment categories which are defined as follows: 0, incomplete exam; 1, no findings; 2, benign; 3, probably benign; 4, suspicious; 5, highly suggestive of malignancy; and 6, known biopsy-proven cancer. Because the database lacks reliable pathological confirmation of malignancy, we assigned all images with BI-RADS 1 and 2 as negative; BI-RADS 4, 5 and 6 as positive; and excluded 12 patients and 23 images with BI-RADS 3 since this assessment is typically not given at screening. We split the dataset 70:30 into training and test sets at the patient-level while maintaining the same ratio of positive and negative images. The total numbers of images in the training and test sets were 280 from 72 women and 107 from 31 women, respectively. We used the same processing steps on the INbreast images as for the CBIS-DDSM images.

#### Effectiveness and efficiency of transfer learning

Although the INbreast database contains ROI annotations, they were ignored to test the transferability of the whole image classifier across different mammography platforms and databases. The four best performing models (See Tables 2 and 3) were directly fine-tuned on the INbreast training set and evaluated by computing per-image AUCs on the test set. Adam^42^ was used as the optimizer and the learning rate was set at 10^−5^. The number of epochs was set at 200 and the weight decay at 0.01. All four models achieved an AUC of 0.95 (Table 4). The ensemble model based on averaging the four best models improved the AUC to 0.98 with a corresponding sensitivity of 86.7% and specificity of 96.1% (Fig. 3b).

**Table 4:** Transfer learning efficiency with different training set sizes assessed by the per-image AUC on the INbreast test set.

#Patients	#Images	Resnet-Resnet	Resnet-VGG	VGG-VGG	VGG-Resnet
20	79	0.92	0.88	0.87	0.89
30	117	0.93	0.94	0.93	0.90
40	159	0.93	0.95	0.93	0.93
50	199	0.94	0.95	0.94	0.93
60	239	0.95	0.95	0.95	0.94
72 (All)	280	0.95	0.95	0.95	0.95

We also sought to determine the minimum amount of data required to fine-tune a whole image classifier to a satisfactory level of performance, to guide future studies in minimizing the resource intensive process of obtaining labels. Training subsets with 20, 30, 40, 50 and 60 patients were sampled for fine-tuning and model performance was evaluated using the same test set (Table 4). With as few as 20 patients or 79 images, the four models already attained AUCs between 0.87 and 0.92. The AUCs quickly approached the maximum as the training subset size increased. These results suggest that the intensive part of learning is to recognize the shapes and textures of the benign and malignant ROIs and normal tissues, and that adjusting to different intensity profiles found in different mammography datasets may require much less data. Importantly, these results clearly demonstrate that the end-to-end training approach can be successfully used to fine-tune a whole image classifier using additional small training sets with image-level labels, greatly reducing the burden of training set construction for multiple different mammography platforms.

## Discussion

This study shows that accurate classification of screening mammograms can be achieved with a deep learning model trained in an end-to-end fashion that relies on clinical ROI annotations only in the initial stage. Once the whole image classifier is built, it can be fine-tuned using additional datasets that lack ROI annotations, even if the pixel intensity distributions differ as is often the case for datasets assembled from heterogeneous mammography platforms. These findings indicate that deep learning algorithms can improve upon classic commercial CAD systems, such as iCAD SecondLook 1.4 and R2 ImageChecker Cenova 1.0, that are not deep learning based and have been reported to attain an average AUC of 0.72^6^. Our all convolutional networks trained using an end-to-end approach have highly competitive performance and are more generalizable across different mammography platforms compared with previous deep learning methods that have achieved AUCs in the range of 0.65–0.97 on the DDSM and INbreast databases, as well as in-house datasets^12^. Two recent studies reported that a new commercial CAD system, Transpara 1.4.0, attained an AUC of 0.89 when used to support radiologists^16^ and 0.84 in standalone mode^15^. This commercial CAD used CNNs trained using the lesion annotations from 9000 mammograms with cancer to generate scores at the patch level; the scores for all detected regions were then combined into a score at the examination level. To our knowledge, the commercial CAD cannot easily be fine-tuned on different mammography datasets without lesion annotations. Our approach has the advantage of requiring only image-level labels for fine-tuning once the whole image classifier is built to facilitate scaling to larger datasets and transferring to new mammography systems as they rapidly evolve.

Two recent studies^45,46^ developed deep learning based methods for breast cancer classification using film and digital mammograms, which were end-to-end trainable. Both studies used multi-instance learning (MIL) and modified the whole image classifier cost functions to satisfy the MIL criterion. In contrast to our approach, neither study utilized ROI annotations to train the patch classifiers first and the AUCs were lower than reported in this study. We found that the quality of the patch classifiers is critical to the accuracy of the whole image classifiers. This was supported by two lines of evidence. First, the whole image classifier based on the S10 patch set performed far better than the one based on the S1 patch set because the S10 patch set contained more information about the background than the S1 patch set. Second, it took much longer for the VGG16-based whole image classifiers to achieve the same performance as the Resnet50-based classifiers because the VGG16 was less accurate than Resnet50 in patch classification.

We also found that the accuracy of whole image classification was improved by sampling more or larger patches to include neighboring regions around the ROI and additional background regions. However, the computational burden increases linearly with the number or size of patches sampled and the performance gain may quickly diminish. Using larger patches can decrease the signal-to-noise ratio, as indicated by the lower patch classification accuracy using the S1g vs. S10 patch sets. Using larger patches also requires higher GPU memory, which may limit network choices. The saliency map analysis showed that our whole image networks were able to correctly identify the ROIs and use the information therein to predict cancer. It also showed that classification errors typically occurred in difficult cases, such as benign lesions with malignant features, or malignant lesions that were difficult to distinguish from background. Further research is needed to investigate how to sample local patches more efficiently, perhaps by augmenting the training data with difficult cases and focusing on the patches that are more likely to be misclassified. This could help overcome the computational burden of training more accurate classifiers.

Although the VGG-based image classifiers were more prone to overfitting and required longer training, the performance of VGG-based and Resnet-based image classifiers was comparable. The fact that the ensemble model performed better than any of the individual models also suggests that the VGG-based and Resnet-based classifiers can complement each other. Moreover, the VGG16 (without the two FC layers), with 15 million weight parameters, is a much smaller network than the Resnet50, with 24 million weight parameters. Having fewer parameters reduces memory requirements and training time per epoch, which is important when computational resources are limited. The Resnet is a more recently developed deep learning method, which is enhanced by shortcuts and batch normalization, both techniques that may help the network train faster and generalize better. The same techniques can be used in the VGG-based networks as well in future work, which may improve the VGG-based classifiers.

This study had some limitations. Mammograms were downsized to fit the available GPU (8 GB). As more GPU memory becomes available, future studies will be able to train models using larger image sizes, or retain the original image resolution without the need for downsizing. Retaining the full resolution of modern digital mammography images will provide finer details of the ROIs and likely improve performance. Although the CBIS-DDSM dataset included pathological confirmation of all cancer diagnoses, the INbreast dataset did not. Therefore, we used the radiologists’ BI-RADS assessments to assign labels to the images in the INbreast dataset, which has the limitation of reproducing radiologists’ impressions instead of discovering new characteristics of malignant lesions. It would be of interest in future work to include interval breast cancers that were missed by radiologists, to help train algorithms to detect more subtle signs of malignancy that may not be visually apparent. Finally, the CBIS-DDSM and INbreast datasets were not nationally representative samples and performance metrics in these datasets are not directly comparable to national estimates of radiologists’ sensitivity and specificity. Future direct comparisons between algorithms and radiologists will be facilitated by public sharing of the code and greater availability of representative benchmarking datasets.

In conclusion, our study demonstrates that deep learning models trained in an end-to-end fashion can be highly accurate and potentially readily transferable across diverse mammography platforms. Deep learning methods have enormous potential to further improve the accuracy of breast cancer detection on screening mammography as the available training datasets and computational resources expand. Our approach may assist future development of superior CAD systems that could be used to help prioritize the most suspicious cases to be read by a radiologist, or as an automatic second reader after making an initial independent interpretation. Our end-to-end approach can also be applied to other medical imaging problems where ROI annotations are scarce.

## Supplementary information

Dataset 1

## Data Availability

A preprint version of this article is available at: https://arxiv.org/abs/1708.09427.
