# Supplementary material for: Deep Learning to Improve Breast Cancer Detection on Screening Mammography
Source: Sci Rep. 2019 Aug 29;9:12495. doi: 10.1038/s41598-019-48995-4 (PMC6715802; doi:10.1038/s41598-019-48995-4)
Supplement: Supplementary file 1 — Dataset 1 [file 41598_2019_48995_MOESM1_ESM.docx]

**Deep Learning to Improve Breast Cancer Detection on Screening Mammography**

**Li Shen, Laurie R. Margolies, Joseph H. Rothstein, Eugene Fluder, Russell McBride, and Weiva Sieh**

Table S1: Per-image AUC difference 95% confidence intervals of augmented inference using the four flipped images (See main text) vs. single inference using the original image. All patch classifiers are based on the S10 set.

| Patch classifer | Block1 | Block2 | AUC difference [95% CI] |
| --- | --- | --- | --- |
| Resnet50 | [512-512-2048] x 1 | [512-512-2048] x 1 | 0.018 [0.0015, 0.034] |
| Resnet50 | [512-512-1024] x 2 | [512-512-1024] x 2 | 0.013 [-0.0029, 0.028] |
| Resnet50 | [256-256-256] x 1 | [128-128-128] x 1 | 0.010 [-0.0052, 0.026] |
| Resnet50 | 256 x 1 | 128 x 1 | 0.0041 [-0.0049, 0.013] |
| VGG16 | 512 x 3 | 512 x 3 | 0.013 [-0.0024, 0.029] |
| VGG16 | 256 x 1 | 128 x 1 | 0.018 [0.0053, 0.030] |
| VGG16 | 128 x 1 | 64 x 1 | 0.016 [0.0027, 0.030] |
| VGG16 | [512-512-1024] x 2 | [512-512-1024] x 2 | 0.033 [0.017, 0.049] |
